# Adaptation and validation of indicators concerning the sterilization process of supplies in Primary Health Care services^1^

**DOI:** 10.1590/0104-1169.3518.2536

**Published:** 2015

**Authors:** Isis Pienta Batista Dias Passos, Maria Clara Padoveze, Camila Eugênia Roseira, Rosely Moralez de Figueiredo

**Affiliations:** 2 MSc; 3 PhD, Professor, Escola de Enfermagem, Universidade de São Paulo, São Paulo, SP, Brazil; 4 PhD, Associate Professor, Departamento de Enfermagem, Universidade Federal de São Carlos, São Carlos, SP, Brazil

**Keywords:** Primary Health Care, Sterilization, Nursing, Indicators of Health Services, Validation Studies as Topic

## Abstract

**OBJECTIVES:**

to adapt and validate, by expert consensus, a set of indicators used to assess the sterilization process of dental, medical and hospital supplies to be used in PHC services.

**METHOD:**

qualitative methodological study performed in two stages. The first stage included a focal group composed of experts to adapt the indicators to be used in PHC. In the second stage, the indicators were validated using a 4-point Likert scale, which was completed by judges. A Content Validity Index of ≥ 0.75 was considered to show approval of the indicators.

**RESULTS:**

the adaptations implemented by the focal group mainly referred to the physical structure, inclusion of dental care professionals, inclusion of chemical disinfection, and replacement of the hot air and moist heat sterilization methods. The validation stage resulted in an index of 0.96, which ranged from 0.90 to 1.00, for the components of the indicators.

**CONCLUSION:**

the judges considered the indicators after adaptation to be validated. Even though there may be differences among items processed around the world, there certainly are common characteristics, especially in countries with economic and cultural environments similar to Brazil. The inclusion of these indicators to assess the safety of healthcare supplies used in PHC services should be considered.

## Introduction

The quality of sterilization and disinfection is critical for the control and prevention of Health-Associated Infections (HAIs), since infections can be acquired due to poor processing. Studies show the need for appropriate disinfection and sterilization of medical equipment and instruments^(^ 1 ^-^ 3 ^)^.

Even though the actions performed within Primary Health Care (PHC) services employing sterilizable devices are not technically complex, the disinfection of these devices in PHC services is a very complex activity, the main objective of which is to avoid adverse events related to the use of these devices. Disinfection requires operational ability and expertise on the part of the professionals involved^(^ 4 ^)^. The risks inherent to inappropriate processing are related to the potential transmission of microorganisms that cause infection, to the toxicity of the disinfectant products used in the process, and potential adverse events related to residue from immunological material transmitted from one patient to another^(^ 5 ^)^.

The prevention and control of infection in PHC units have been overshadowed by news highlighting HAIs in hospital facilities, in addition to the few studies addressing this topic in extra-hospital environments^(^ 6 ^)^. Nevertheless, the same criteria and training required in the Sterile Processing Departments (SPDs) of hospital facilities should be followed at the PHC level^(^ 7 ^)^.

Due to the increase and diversification of extra-hospital care services and to the pressing need to establish HAI control practices in different environments, it is essential to provide a standardized procedure to assess the quality of processing in SPDs.

Therefore, this study's objective was to adapt and validate a set of indicators to assess the sterilization processing of dental and medical articles through expert consensus, to be used within PHC services^(^ 8 ^)^.

## Method

This is a methodological study of adapting and validating an instrument, conducted with experts to assess measures in the health field (indicators of quality of processing). The validation of an instrument through the analysis of its psychometric qualities aims to objectify and improve its use^(^ 9 ^)^, because it strengthens the reliability of results. The object of study is a set of indicators developed and validated to assess the processing of dental, medical and hospital devices within hospital facilities. This is the first instrument developed for this purpose with methodology available both in Brazil and outside of Brazil^(^ 8 ^)^. It contains ten indicators addressing the stages of sterilization processing in the health field, including structure, process and result. Each indicator contains components to be assessed and presents how to make the assessment (inspection, record, and interviews), as well as a formula to calculate compliance. The indicators are numbered according to the type of process to which they are related: cleaning, preparation/packaging, and sterilization^(^ 8 ^)^.

In the first stage, the study's population was composed of six experts and the second stage included 11 judges, who assessed content validity.

### First stage - Adaptation of indicators for PHC

This stage was implemented using a focal group. Each item of the original instrument was presented and discussed and after consensus was reached among the experts, the instrument would be: maintained as it was; content would be maintained though with new redaction; the item would be excluded; or a new item would be included. The final version of the instrument adapted to PHC services presented a total of nine indicators. Resolution No. 15 from March 15 2012, Joint Executive Board (RDC)^(^ 10 ^)^ was published after the focal group had been conducted; some adaptations were required afterwards.

### Second stage - Indicators' content validity

This stage included consensus on the part of experts (judges). When these judges were selected, their experience and high level of knowledge regarding the subject was considered. The judges were chosen so that there would be representatives of both the processing field and from PHC. A total of 11 judges participated in this stage. They were 49.9 years old on average and six (54.5%) had more than 30 years of experience. All of them were experts in nursing; seven (63.6%) had a Master's degree and six (54.5%) a Doctoral degree. Seven (63.6%) teach in undergraduate programs and all have provided nursing care for at least four years. Seven (63.6%) judges published papers in the last five years in periodicals addressing the prevention and control of HAIs. After an initial contact via telephone or email, the printed material was sent by mail.

The adapted instrument was composed of a four-point, psychometric, Likert scale: (1) does not address the attribute; (2) addresses the attribute but requires considerable changes or new redaction; (3) addresses the attribute but requires minimum changes; and (4) addresses the attribute. In the case where there was some item for which minimum agreement was not reached after the judges' assessments, we considered the possibility of adjusting the item based on the judges' suggestions. A Content Validity Index (CVI) of 0.75 was considered in order to attain 75% consensus^(^ 11 ^)^. Options (3) and (4) were totaled to validate the components and options (1) and (2) were totaled to establish their exclusion. To validate the instrument as a whole, the mean of the proportions of the items deemed relevant by the judges (sum of responses 3 and 4) was computed.

The study was approved by the Institutional Review Board at the *Centro Universitário Central Paulista*, (No. 054/2011) and the participants signed free and informed consent forms.

## Results

### First stage - Adaptation of indicators for PHC

In the phase of the instrument adaptation, the indicators concerning physical structure showed a need for adaptations concerning the physical area; that is, the judges did not consider it mandatory that there be two isolated areas per physical structure (dirty and clean areas), provided that the minimum requirement of there being a technical barrier was met. The inclusion of dental health professionals in the processing (oral health technicians and auxiliaries) was recommended due to the characteristics of PHC in Brazil, in which dental care is relevant. In regard to the process' indicators, there was the inclusion of chemical disinfection and moist heat sterilization only, while alternative thermo-sensitive sterilization methods were not considered. One indicator concerning the assessment of conditions of conservation of disinfected instruments' packaging was added to the result indicators . 

The original indicators were classified into three categories according to the main stages of processing, i.e., cleaning (C), preparation/packaging (P), and sterilization/storage/distribution (S). In the adapted version, the indicators concerning preparation, packaging, sterilization, storage, and distribution were grouped together because these processes can be performed in the same area. The result of the adaptation concerning the cleaning (C) indicator generated the following components: 26 concerning structure (C1); 15 concerning process (C2); one for results (C3); and one concerning occupational biological risk (C4). The results concerning the adaptation of the Indicators of Preparation and Sterilization (PS) generated the following assessment components: 20 components addressing structure (PS5); 35 concerning process (PS6); and three addressing results (PS7, PS8, PS9).

### Second stage - Indicators' content validity

The mean CVI concerning the instrument's general assessment was 0.96. Among the 101 components assessed, 96 obtained satisfactory CVI (≥0.75) in assessment criteria 3 and 4. (Table 1).

**Table 1 -:** Content Validity Index (CVI) obtained by expert consensus. São Carlos, SP, Brazil, 2013

Assessment Criteria	CVI by Set of Indicators									
	Cleaning					Preparation /Sterilization				
	C1	C2	C3	C4		PS5	PS6	PS 7	PS 8	PS 9
1. Does not address the attribute	0.04	0.04	0.00	0.10		0.05	0.01	0.00	0.00	0.00
2. Addresses the attribute	0.02	0.06	0.00	0.00		0.03	0.05	0.00	0.00	0.00
3. Addresses the attribute but requires considerable changes or new redaction	0.18	0.12	0.20	0.30		0.14	0.11	0.00	0.00	0.00
4. Addresses the attribute	0.76	0.79	0.80	0.60		0.78	0.84	1.00	1.00	1.00
Final CVI*	0.94	0.90	1.00	0.90		0.92	0.95	1.00	1.00	1.00

* Final CVI = sum of the CVI of criteria 3 and 4.

**Figure 1 -:**
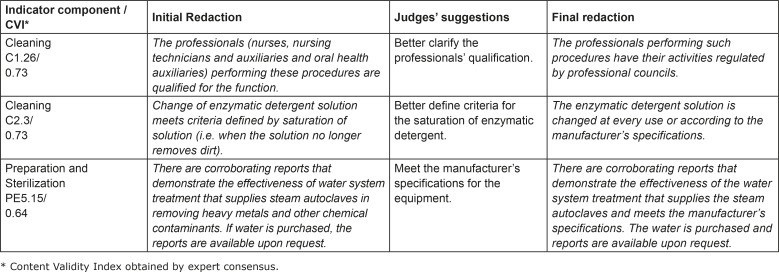
Initial redaction, judges' suggestion, and final redaction of components of indicators and respective CVI. São Carlos, SP, Brazil, 2013

Some components obtained a CVI below 0.75 and a new version of these components was proposed, taking into account the judges' suggestions. The initial and final redactions of these components, including the judges' suggestions, are presented in Figure 1.

Because RDC No. 15^(^ 10 ^)^ was applied, the understanding was that SPDs in PHC services are classified as Class I. Based on this assumption, some components were excluded when they referred to the process of instruments/devices of complex design/construction. These include: special nozzles for tubular items; water guns for cleaning tubular items and complex design items, brushes with different diameters used for tubular items; and ultrasonic washers. It is worth noting that in regard to inhalation masks, even though these need to be disassembled for processing, they are not items of complex design, and, therefore, a specific protocol for disassembling them was not considered necessary.

Two components (structure and process), which refer to event-related sterility, were included in indicators PS5 and PS6. These components were:* There is a clearly described assessment plan concerning the integrity of the processed item's packaging (*PS5.21*) *and *The packages of processed items are assessed in regard to their integrity (*PE6.36*).*

The indicators and instructions for applying the instrument are contained in the Operational Manual available at: www.cve.saude.sp.gov.br/htm/ih/ih_doc.html.

## Discussion

The construction and validation of indicators, as well as an assessment of practices to control HAIs through the use of indicators, has increased; however, research addressing this subject in Brazil is still incipient and not homogenous^(^ 12 ^)^.

Consensus was easily achieved during the adaptation phase. Doubts identified by the group were clarified through consultation of related literature. One of the main adaptations concerning structure was the adaptation of the physical area, where a single area (clean and dirty), not necessarily divided by a physical structure, is possible as long as there is a technical barrier. This recommendation, provided for Class I SPDs, was based on RDC No. 15, where a technical barrier is defined as "a set of behavioral measures, taken on the part of healthcare workers in the absence of physical structures, intended to prevent cross-contamination between the dirty and clean environments."^(^ 10 ^)^. This concept implies the need to define areas so that clean items are not handled on the same surfaces where dirty items are handled, thereby decreasing microbial load even when there are no structural barriers such as walls or dividers. 

Chemical disinfection, more frequent in PHC services than in hospital facilities, mainly occurs in the processing of aerosol kits. These kits, still frequently used in PHC facilities in the treatment respiratory tract disorders, are a challenge for sterilizing processing, especially tubular extensions, and for this reason deserve special care due to the difficulty of cleaning lumens^(^ 13 ^)^. There is no consensus among experts or evidence in the literature regarding the need to disinfect the extension's internal lumen, since it is not in direct contact with patient secretions. The disinfection of the internal lumen implies the need for carefully drying it after processing to avoid residual fluid and the growth of microorganisms. Nevertheless, the availability of medical compressed air systems, inert gas, or filtered air in processing rooms within Brazilian PHC services is still a challenge. Sodium hypochlorite is still the most commonly used disinfectant in PHC, especially due to its low cost, despite controversy in the literature regarding its indication and, if indicated, the concentration to be used^(^ 3 ^,^ 14 ^)^.

It is also important to assess the integrity of how items are packaged that were already processed, because they should be individually wrapped in sealed plastic after drying. Packaging is intended to avoid recontamination during storage and the handling of disinfected items before reuse^(^ 14 ^)^.

The restricted use of hot air sterilization is provided in the Brazilian guidelines^(^ 10 ^)^. It is believed that this specification was restated in accordance with the literature that shows that the use of ovens is still very common in healthcare services. One study reports that 15 out of 44 hospitals in Goias, Brazil still use Pasteur ovens for sterilization. Such a method has fallen into disuse due its operational difficulty and technological advancements beyond it^(^ 2 ^)^. Most hospitals did not apply physical, chemical or biological controls for the sterilization cycles. Another study, performed in cities in the interior of São Paulo, reports its use in dental care facilities, in which only 6% of the ovens indicated the temperature that was attained inside the equipment and had thermostats to maintain the desired temperature^(^ 15 ^)^.

Most comments and suggestions were incorporated in the validation phase.

Change of redaction concerning the qualification of professionals processing items in the SPDs of PHC services was necessary to comply with RDC No.15^(^ 10 ^)^, which provides that all processing activities be performed by professionals whose activity is regulated by professional councils. 

There are oral care professionals (oral care technicians and auxiliaries) processing items in PHC services. One study conducted in PHC units in a city in the interior of the state of São Paulo, Brazil, shows that the nursing staff was responsible for the processing of items in almost all the units (97%); only in one unit (2.9%) was the professional responsible for this task a dental professional^(^ 7 ^)^. Another study shows that for most of the services in the city of Goiania, GO, Brazil, the professionals responsible for the processing of dental items were oral health auxiliaries (48%) or dental hygiene technicians (21%), while in the remaining services, this task is performed by workers without specific training in the health field (21% were auxiliaries with practical experience and 10% were general services assistants^(^ 16 ^)^. The processing of items performed by personnel without technical qualification may compromise the quality of care delivery^(^ 2 ^)^.

The same study reports that the dental office's environment was used to process the items in 55% of the cases. It is ideal that both the nursing and dental staff are connected, so that processing is centralized. There is greater rationalization of work, optimization of human resources and material, in addition to greater safety for both patients and workers, when there is a centralized system^(^ 17 ^)^.

Publications addressing the processing of items in the dental field have advanced, which shows a concern with the topic^(^ 16 ^,^ 18 ^)^. There is a need, though, for further studies so that these processes may be standardized.

Recommendations concerning replacing the enzymatic detergent used in cleaning items state that the detergent must be replaced frequently so that the solution does not become saturated with organic matter, which decreases its efficacy^(^ 19 ^-^ 20 ^)^. In this study, the recommendation is to follow the manufacture's specifications because, in Brazil, enzymatic detergent manufacturers must comply with RDC No. 55^(^ 21 ^)^, indicating on the product's label that reusing the solution may impair its cleaning efficiency. 

The quality of the water used in autoclaves was questioned. According to the manufacturers, potable water is not indicated to supply autoclaves. This water contains organic and inorganic particles, some pesticides and disinfectant that may impair the equipment^(^ 19 ^)^. Current Brazilian drinkability standards do not ensure the removal of such particles. Therefore, the recommendation is to follow manufacturer specifications^(^ 10 ^)^. The use of distilled water or water purified by reverse osmosis filters is suitable to avoid the use of water containing undesirable elements. 

The judges questioned the suggestion to use non-woven fabrics (NWF) to dry the items. There is difficulty in the context of PHC services; washing and drying reusable fabrics and disposable NWF ease the process. Fabrics can be used if there is a possibility to launder the fabrics (e.g., compresses).

In the context of the PHC services in the city under study, the processed items are of simple design. In the dental field, however, there is a 1mm diameter suction tube that is classified, according to current legislation, as an item of complex design. To ensure the quality of care and maintain the classification of SPDs within PHC services as class I, this single product may be replaced by a single-time use, disposable item.

Current criteria regarding the expiration date of sterilization processes recommend changing to event-related sterility as opposed to time-related sterility. The subject was suggested by one of the judges and consultation of the literature shows that sterility should not be time-related but subjected to the occurrence of some related event that may compromise the package's integrity. Sterility is related to the package, seal, environmental conditions, items' design, and handling^(^ 5 ^,^ 19 ^)^. One study verified that, after two years, 100% (152) of the sterilized and stored packages, which suffered no adverse events, were sterile^(^ 22 ^)^. Another study concluded that even after exposure to the microorganism *Serratia marcescens*, an item with a safe microbial barrier remained sterile after 180 days^(^ 23 ^)^. Event-related sterility is safe and should, therefore, replace time-related expiration^(^ 22 ^)^.

## Conclusion

The instrument was satisfactorily adapted and validated to be used in PHC services in the three dimensions of indicators: structure, process and results. The main differences between hospital processing and PHC processing are the physical structure (concept of technical barrier), inclusion of dental care professionals, inclusion of components for chemical disinfection and recommendation of the use of event-related sterility. 

Some topics deserve deeper analysis, such as the definition of complexity of dental health supplies used in PHC services, the quality of water supplied to autoclaves, criteria of enzymatic detergent saturation and chemical disinfection. Potential limitations of this study involve the fact that all the judges were RN and originated from only the Southeast and Midwest regions of Brazil.

Even though content validity is key for the development and adaptation of new measures, it has a subjective nature and additional psychometric measures need to be applied. Thus, we suggest further studies addressing other validation measures to assess the applicability of the instrument adapted in this study.

We highlight the importance of this instrument being validated for the assessment of the quality of sterilization processes of healthcare supplies specifically for PHC services. The appropriate processing of healthcare supplies is one of the primary measures to advance HAI preventive actions. Hence, this study presents a relevant contribution to the improvement of the quality of care delivered in PHC services.
